# Re-Evaluating the Geological Evidence for Late Holocene Marine Incursion Events along the Guerrero Seismic Gap on the Pacific Coast of Mexico

**DOI:** 10.1371/journal.pone.0161568

**Published:** 2016-08-29

**Authors:** Thomas A. Bianchette, Terrence A. McCloskey, Kam-biu Liu

**Affiliations:** Department of Oceanography and Coastal Sciences, College of the Coast and Environment, Louisiana State University, Baton Rouge, Louisiana, United States of America; Institute of Tibetan Plateau Research Chinese Academy of Sciences, CHINA

## Abstract

Despite the large number of tsunamis that impact Mexico’s Pacific coast, stratigraphic studies focusing on geological impacts are scanty, making it difficult to assess the long-term risks for this vulnerable region. Surface samples and six cores were taken from Laguna Mitla near Acapulco to examine sedimentological and geochemical evidence for marine incursion events. Sediment cores collected from behind the beach barrier are dominated by intercalated layers of peat and inorganic sediments, mostly silt and clay, with little or no sand. Sand- and shell-rich clastic layers with high levels of sulfur, calcium, and strontium only occur adjacent to the relict beach ridge remnants near the center of the lagoon. With the exception of one thin fine sand layer, the absence of sand in the near-shore cores and the predominance of the terrigenous element titanium in the inorganic layers, evidently eroded from the surrounding hillslopes, suggests that these large-grained intervals do not represent episodic marine incursions, but rather were likely formed by the erosion and redeposition of older marine deposits derived from the beach ridge remnants when water levels were high. These results do not support the occurrence of a large tsunami event at Laguna Mitla during the Late Holocene.

## Introduction

Mexico’s Pacific Coast is frequently affected by tsunamis, many of which can cause marine inundations reaching many kilometers inland and result in significant societal damages and geomorphological changes [1, 2]. Globally, many studies have examined sedimentary units deposited via historic or prehistoric tsunami run-ups in order to identify their sedimentological characteristics and spatial extent [3–7]. Studies along Mexico’s Pacific coast have used the presence of sand layers in sediments to identify marine intrusions from tsunamis [8, 9]. To develop accurate risk assessments for this economically vibrant region, additional work must be conducted to establish the long-term (multi-centennial to -millennial) record, with the aim of detecting geological imprints (e.g., sedimentological and/or geochemical signatures) of historical tsunamis to better understand the origins and magnitudes of older events in the sedimentary record. The necessity for this research is especially true for the coastal state of Guerrero, which has experienced at least 48 tsunamis since AD 1732 [1]. Located near the Guerrero Seismic Gap, which has not experienced a large earthquake since 1911 [10–12], this tectonically-active area is due for a major event, possibly of Mw 8.1–8.4 strength [12].

Recent studies from Laguna Mitla, a large backbarrier lagoon along the coast of Guerrero, suggest that a major tsunami impacted this coastal zone ~3400 years ago [12–14]. The geological evidence comes from a ~60 cm thick layer dominated by sand enriched in Na and Sr, overlaid by a ~45 cm thick layer of dark-blue to blue-grey laminated clayish silt occurring from a depth of 300–195 cm in a sediment core extracted from the landward, or back side, of the lagoon [12]. The sand interval was interpreted as having been deposited by an extreme marine incursion, while the overlying blue clay was interpreted as representing a marine-dominated setting [12]. The posited tsunamigenic origin of this deposit [13], if confirmed, would imply that the Guerrero coastal zone, including the major city of Acapulco, is highly vulnerable to the devastating impacts of tsunami activity. In this paper, we present geochemical and sedimentological data from six new cores taken from Laguna Mitla to reconstruct the Holocene depositional history of the site itself, with the objective of evaluating the evidence for marine incursions. Such a re-examination of prehistoric tsunami occurrence is vital for accurately assessing the hazard risks for this coastal region.

## Regional Setting and Study Site

**Fig 1:**
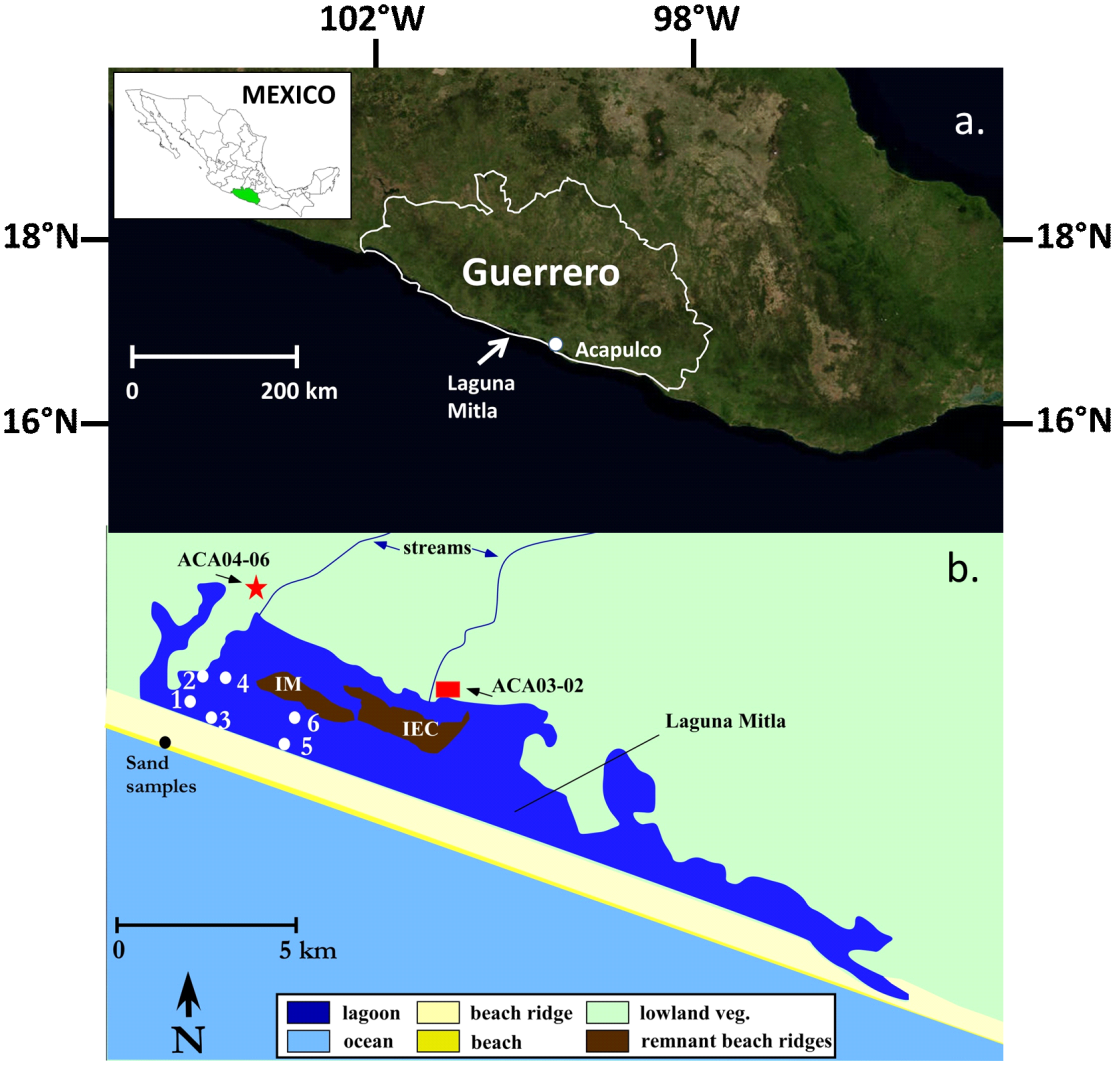
Study site. Location of Guerrero, Laguna Mitla, and Acapulco (a.) along Mexico’s southern Pacific coast (inset). A satellite image (USGS National Map Viewer) of Laguna Mitla is shown (b). A ~60 cm thick, predominantly sand unit attributed to a marine event was discovered in a sediment core (ACA04-06) extracted 5 km from the coast at the location marked with a red star [12]. Sedimentary evidence of a marine intrusion was also reported from a core (ACA03-02) extracted 4 km from the coast at the location marked with a red square [14]. The six coring locations discussed in this paper are marked by white dots. Exposed relict beach ridge remnants Isla Magueyes (IM) and Isla El Conejo (IEC) are labeled.

Guerrero is located along the southern section of Mexico’s Pacific coast (Fig 1). The Sierra Madre del Sur form southwestern Mexico’s ‘spine,’ with elevations surpassing 3,500 m in Guerrero’s interior. The rivers and ephemeral streams that drain the uplands often terminate in coastal lagoons, typically elongated coast-parallel lagoons trapped behind solid beach ridges [15]. When lagoon water level rises during wet periods, pressure along the tidal inlet opening can erode and eventually blowout the beach, partially draining the lagoon [15]. The climate along the coast is monsoonal, with the majority of the annual precipitation (~130 cm) falling during the wet season from May to November [16], which is also the active season for TCs [17].

The Middle America Trench, which marks the subduction of the Cocos plate beneath the continental North American plate, lies ~70 km west-southwest of Laguna Mitla beyond a narrow continental shelf, resulting in significant earthquake and tsunami activity for the area [18].

The Guerrero Seismic Gap covers most of Guerrero’s coastline, extending offshore to the Middle America Trench. The seismic model for this area suggests periods of interseismic (long-term) subsidence interrupted by coseismic uplifts ([13] and references therein). Recent studies have also documented the existence of regional slow slip events [19]. The long-term subsidence, estimated at 0.3–0.4 cm per year, overwhelms the coseismic uplift, as shown by the lack of marine terraces and wave-cut notches along the coast [13].

Laguna Mitla, located 40 km west of Acapulco (Fig 1), is an elongated, shore-parallel lagoon, approximately 22 km long and 4 km wide, with water depths of ~0.5–1 m along the western end. An inactive tidal inlet is located along the lagoon’s southeast corner [20]. The lagoon is classified as oligohaline, with a salinity level of ~3.5 ppt [21]. Runoff from the surrounding hillside is the principal source of inorganic sediments [22]. A ~15 m wide ephemeral stream sourced in the adjacent highlands enters the lagoon’s northwestern edge, nearest our study sites. Lagoon currents are strong enough near the stream to resuspend surface sediments along the western side. Land use to the north of Laguna Mitla is primarily agricultural. Wetland vegetation dominated by *Typha* and mangroves surrounds the lagoon. An extensive salt marsh lies along the northwest edge of the site [12, 14]

Laguna Mitla is morphologically categorized as a “barred inner shelf” lagoon, the most common classification along Mexico’s Pacific coast [15]. Formerly a depression along the continental shelf margin, it was formed during the mid-Holocene transgression when sea levels stabilized and coastal beach ridges formed [15]. The beach ridge plain is ~900 m wide with a maximum height of >8 m. A chain of linear islands, including Isla Magueyes (IM) and Isla El Conejo (IEC), located along the lagoon’s midline are likely relict beach ridge segments [12, 14] deposited during a Pleistocene highstand. Such relict beach ridges are a common feature along Mexico’s Pacific coast [15]. Salt pans dot the interior linear islands, and a plantation covers the eastern half of IM [12, 14].

## Methods

In December 2009, six composite cores were taken along two shore-parallel transects from the lagoon’s western end (Fig 1). No permits were required for the described study, which complied with all relevant regulations. Field studies did not involve endangered or protected species. The cores were extracted with a Russian peat borer in overlapping 50 cm segments. Sediments were transferred to pre-cut PVC tubes and wrapped to prevent moisture loss. Cores 1, 3, and 5 were collected along a ~3.3 km shore-parallel transect behind the beach (seaward transect), whereas cores 2, 4, and 6 comprise a parallel transect closer to the relict beach ridges along the mid-rib of the lagoon (landward transect). Core 2 was extracted 0.5 km landward of core 1 and near the unnamed ephemeral stream. Cores 4 and 6 were extracted 0.5 km and 3.3 km east of core 2, respectively, and near Isla Magueyes. Surface samples were collected from the beach and from lagoon bottom mud surrounding each core site to determine modern sedimentological characteristics. All material was transported to the Global Change and Coastal Paleoecology Laboratory, Louisiana State University, and stored in a refrigerated room (4°C).

Cores were described lithologically and subjected to loss-on-ignition (LOI) analysis [23] at one cm resolution. Samples were dried overnight at 105°C, and burned for one hour at 550°C and 1000°C to determine water (% wet weight), organics (% dry weight), and carbonate (% dry weight) contents, respectively. Seventeen organic (plant/wood) samples were sent to the National Ocean Sciences Accelerator Mass Spectrometry Laboratory (NOSAMS) at the Woods Hole Oceanographic Institution (WHOI) for AMS radiocarbon dating. Results (Table 1) were converted to calendar years using Calib 7.0 [24] and the INTCAL 13 calibration curve [25]. Age models were developed for each core based on the median calendar age, as supplied by Calib 7.0, using linear interpolation between pairs and extrapolation to the core tops and bottoms.

**Table 1:** Radiocarbon dating results for cores 2–6. Calib 7.0 [24] and Intcal 13 [25] were used for calibration.

Core	Depth	Lab #	Material	^14^C age	Cal BP (2δ)	Rel area under prob. distribution	Med. prob
2	114	OS-90703	Plant/Wood	1990±30	1879–1997	1	1940
2	197	OS-93080	Plant/Wood	3850±55	4094–4123	0.038	4271
					4144–4418	0.962	
2	242	OS-90697	Plant/Wood	4080±40	4438–4488	0.118	4582
					4497–4655	0.635	
					4667–4707	0.074	
					4756–4811	0.174	
2	316	OS-93065	Plant/Wood	4430±30	4875–5068	0.765	5016
					5109–5123	0.018	
					5169–5172	0.002	
					5180–5275	0.215	
3	175	OS-91548	Plant/Wood	4110±30	4523–4711	0.747	4632
					4753–4814	0.253	
4	111	OS-92322	Plant/Wood	3840±65	4010–4029	0.012	4254
					4082–4423	0.988	
4	198	OS-93067	Plant/Wood	4110±30	4523–4711	0.747	4632
					4753–4814	0.253	
5	2	OS-90761	Plant/Wood	>Modern	N/A	N/A	N/A
5	34	OS-90770	Plant/Wood	1840±45	1629–1655	0.039	1775
					1660–1666	0.005	
					1693–1880	0.955	
5	61	OS-84452	Plant/Wood	2710±110	2491–2602	0.061	2836
					2607–2642	0.019	
					2678–3083	0.896	
					3090–3145	0.022	
					3152–3155	0.001	
5	125	OS-83891	Plant/Wood	3160±30	3273–3284	0.026	3388
					3340–3450	0.974	
5	207	OS-83892	Plant/Wood	4070±30	4440–4486	0.159	4558
					4499–4644	0.704	
					4677–4692	0.015	
					4761–4800	0.122	
5	290	OS-83942	Plant/Wood	4500±30	5046–5205	0.65	5167
					5210–5296	0.35	
5	324	OS-93066	Plant/Wood	4760±30	5333–5348	0.041	5521
					5353–5371	0.029	
					5463–5587	0.93	
5	414	OS-83941	Plant/Wood	6050±35	6795–6990	1	6903
5	475	OS-90763	Plant/Wood	6000±60	6678–6708	0.031	6842
					6712–6984	0.969	
6	127	OS-92903	Plant/Wood	2800±80	2756–3080	0.965	2917
					3093–3113	0.019	
					3122–3141	0.016	

Once unwrapped, the moist sediments were scanned at two cm resolution with a Delta Innov-X handheld X-ray fluorescence (XRF) equipped with a tantalum x-ray tube and a factory calibration (Compton Normalization) to determine the concentration of 27 chemical elements.

Standards NIST 2702 and 2781 were scanned for validation. Here we present the results of four: strontium (Sr), sulfur (S), calcium (Ca), and titanium (Ti) for cores 1–6. Sr, S, and Ca are enriched in seawater or marine sediments and have been widely used in detecting marine intrusion events in coastal sediments [8, 26–30]; whereas Ti, a lithogenic element, is commonly used as an indicator of soil erosion from the watershed [31]. The surface samples were also XRF-scanned in the laboratory. Surface sample concentrations were averaged according to the corresponding coring locations: four samples from near core 1, five near core 2, three near core 3, three near core 4, five near core 5, and one near core 6, in addition to two collected from the coastal beach.

## Results

**Fig 2:**
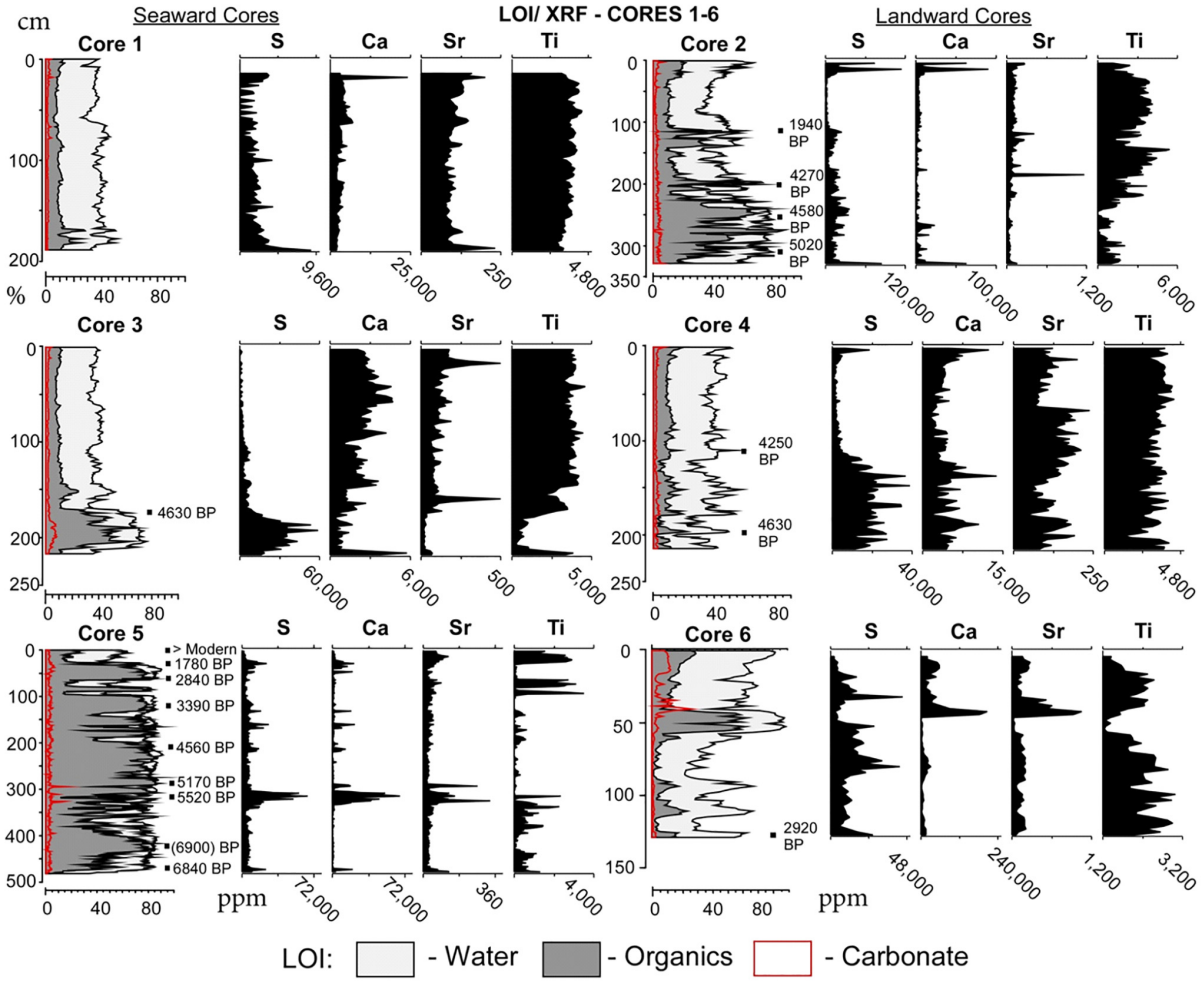
XRF results. Elemental concentrations are paired with LOI results for all cores. Note scales vary between cores.

Cores are generally dominated by inorganic (clay) sediments, marked by low water and organic values (LOI curve, Fig 2). Organic-rich sections consist of either black to dark brown decomposed peat, black to dark brown muddy peat, or dark brown organic clay. Sand is present in only a few cores, usually either as scattered grains or thin, episodic layers. Calibrated calendar dates are shown alongside the LOI curves (Fig 2). Of the seventeen dates, a single stratigraphic reversal occurred, near the bottom of core 5 (6050±35 ^14^C years at 414 cm, 6000±60 ^14^C years at 475 cm). The date at 414 cm was rejected as its inclusion would imply an improbably large change in the sedimentation rate.

### Seaward transect

Core 1 (190 cm) was extracted near Laguna Mitla’s western, seaward edge from a water depth of ~50 cm. Two thin, dark brown organic-rich clay layers occur near the core bottom at 183–177 and 173–170 cm (Fig 2). The remaining sediment is mostly gray to brown clay, with low percentages of water (~35–40%), organic (6–12%), and carbonate (1–3%). Ti concentrations increase slightly up-core. With the exception of large spikes in S and Sr in the basal material and in Ca near the core top, elemental concentrations are fairly stable (Fig 2). Water (24–32%) and organic (~5–8%) percentages are depressed from 64–53 cm in the interval containing clay with fine sand. Small gastropods are scattered near the core top.

Core 3 (218 cm) was retrieved from ~50 cm water depth ~0.5 km east of core 1. A thick peat section from 212–164 cm lies on top of 6 cm of clay. Clay, marked by fairly consistent percentages of water (~30–40%), organic (~7–24%), and carbonate (~1–4%) dominates the remainder of the core (Fig 2). S concentration decreases up-core, while Ti, Sr, and Ca concentrations increase up-core (Fig 2).

Core 5 (479 cm), extracted ~3 km east of core 3 from ~1 m water depth, consists of gray sand throughout the bottom 8 cm. The remaining 471 cm is dominated by black peat with high water (~85%) and organic (~80%) values (Fig 2). The clay occurring from ~400–291 cm has a relatively high concentration of Ti, with the exception of four carbonate-rich layers at 294–292 (14%), 309–308 (8%), 327–325 (13%), and 394–392 cm (6%). These carbonate layers are marked by elevated concentrations of S, Sr, and Ca and low concentrations of Ti (Fig 2). Seven Ti-rich clay layers varying in thickness and composition occur within the otherwise homogenous peat section from 290–0 cm.

### Landward transect

Core 2 (329 cm) was extracted 0.5 km landward of core 1 at ~50 cm water depth. The bottom section of the core (329–196 cm) is a dark, muddy peat containing thin gray clay layers with low water and organic values (Fig 2). The top 196 cm consists of brown to gray clay, with a dark, organic-rich clay section at 145–115 cm. Ti concentrations are elevated in the low-organic clay sections. At 145–144 cm, the concentration of Ti (5,428 ppm) is the transect maximum (Fig 2). Concentrations of S, Ca, and Sr are lower throughout this clay section than in the underlying muddy peat, with the exception of peaks in S and Ca near the core top, which contains scattered gastropod shells.

**Fig 3:**
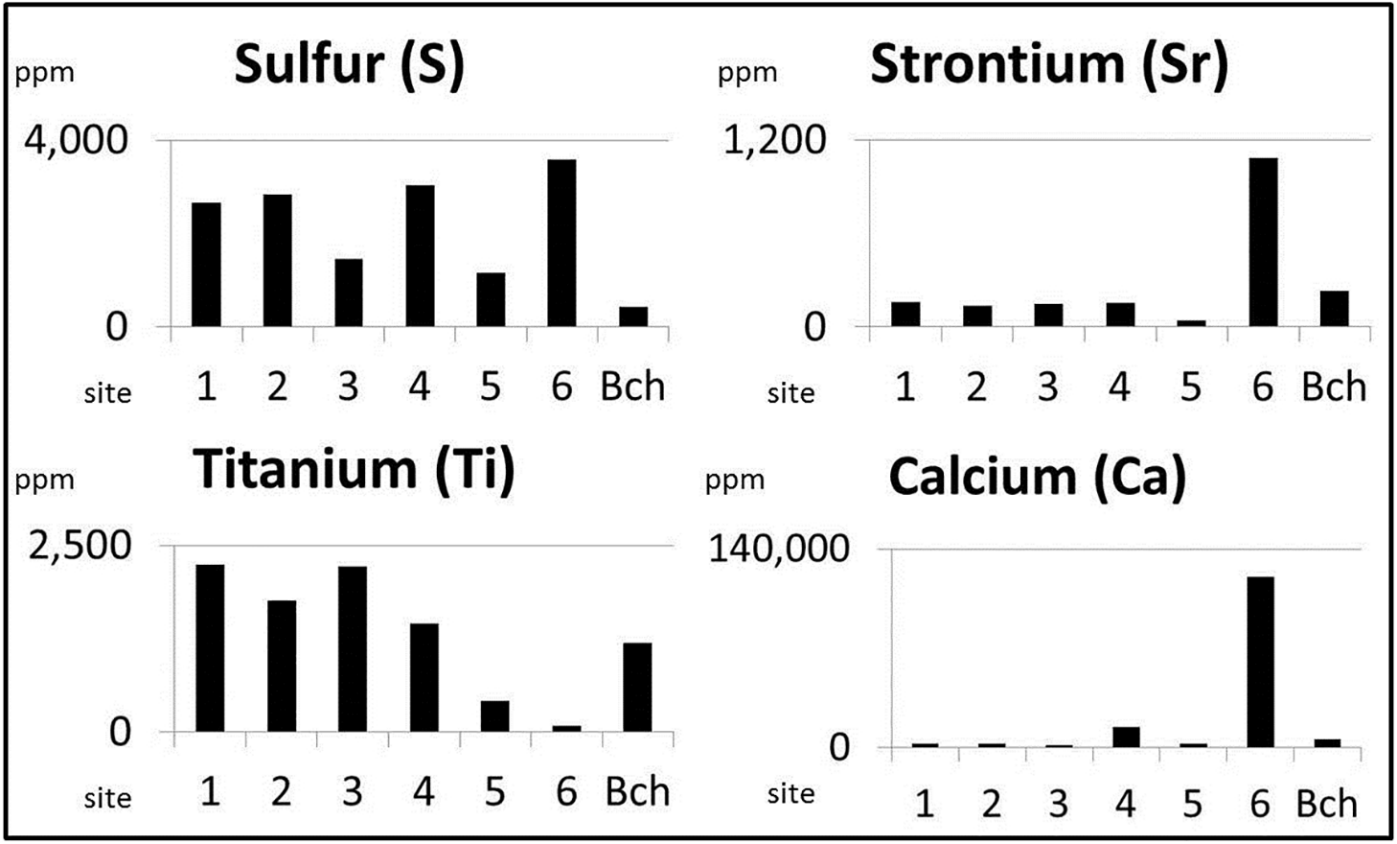
Elemental concentrations of surface samples. Surface samples were collected from the coastal beach and adjacent of the coring sites. Concentrations from surface samples belonging to the same coring site were averaged and plotted. Titanium concentrations decrease eastward across the transects. Concentrations of sulfur, strontium and calcium, commonly-used marine indicators, are higher in the landward sites (2, 4, 6) than in the paired seaward sites (1, 3, 5). Beach samples contain relatively low concentrations of sulfur, strontium and calcium.

Core 4 (216 cm) was extracted 0.5 km east of core 2 at a water depth of ~50 cm. Water and organic percentages, and Ti, Ca, and Sr concentrations are highly variable throughout the bottom section (216–119 cm), which consists of brown to gray clay interlayered with scattered yellow sand layers (Figs 2 and 3). S concentrations are high in this material. The upper 119 cm consists of brown to gray clay with relatively constant water (30–40%), organic (7–10%), and carbonate values (1–2%). Above 119 cm, concentrations of S decrease significantly as Ti increases slightly. Ca concentrations increase near the core top, while Sr concentrations are highly variable throughout the section. Small gastropods are scattered throughout the sediments near the core top.

Core 6 (129 cm) was extracted ~3 km east of core 4 from a depth of ~1 m. An alternating series of dark gray/black sandy peat and coarse gray sand occurs from 129–88 cm, with higher concentrations of Ti, S, Sr, and Ca in the sand layers (Fig 2). A dark gray sand layer from 88–56 cm is marked by low water, organic, and carbonate values (Fig 2), and higher S concentrations. A dark peat occurs from 56–45 cm. The top 45 cm consists of iterations of reddish, gray, and brown clay. A shell hash section at 45–33 cm is marked by a sharp spike in carbonates (32%) and concentrations of Ca and Sr. Ti concentrations are elevated in the reddish clay near the core top.

All surface samples are clay-dominant with the exception of the sample near site 6, which contains some shell hash and shiny, flaky silicates, possibly mica. S concentrations are higher near the landward sites 2, 4, and 6, than at the seaward sites 1, 3, and 5 (Fig 3). Sr concentrations are nearly an order of magnitude higher at site 6 than at any of the other five sites: site 1, 2, 3, 4, and 5. Ca concentrations are also higher near site 6. Ti concentrations are higher near the western sites, sites 1, 2, 3, and 4, and lower near the eastern sites 5 and 6. S, Sr, and Ca concentrations are relatively low in the beach samples (Fig 3).

## Discussion

The chronologies and stratigraphies of the six cores along two transects can be cross-correlated and synthesized to reveal the depositional processes and provenances of the source material, a critical necessity for evaluating the occurrence of prehistoric marine intrusions in this coastal zone.

### Paleoenvironmental reconstruction

The multi-proxy data from core 5 are presented in detail elsewhere [32] to provide a 7000-year reconstruction of the coastal paleoenvironmental evolution and Holocene paleoclimatic changes at Laguna Mitla, and are thus not the main objectives of this paper. However, brief descriptions of regional environmental history and site evolution are presented here to provide a context for understanding the depositional patterns and processes within the lagoon, the focus of this paper. Core 5, the longest and best-dated core, provides a sedimentary record spanning the last 7000 years (Fig 2). At the beginning of the record (~ 6900 years BP), the core site was dominated by *Rhizophora* (red mangrove) [32]. By ~6200 years BP, the rising seas began depositing a mix of offshore clastics and entrained terrestrial sediments, marking the initiation of the beach barrier as documented along Mexico’s Pacific coast [33]. Clastic input was temporally variable due to varying degrees of wave energy from the spatially discontinuous and highly transient beach barrier, perhaps frequently subjected to perturbations from overwash and hydrodynamic processes. This highly dynamic period lasted until the rate of sea level rise slowed at ~5200 years BP [33], at which time the barrier became more stable and consolidated. From ~5200 years BP to present, the beach ridge plain has prograded seaward, and the site has existed as a backbarrier system isolated from the sea by the beach barrier. The build-up and consolidation of the beach barrier increasingly isolated the backbarrier lagoon from the ocean, so that water level at the coring sites has since been essentially controlled by precipitation, rather than sea level. Throughout the last 5000 years, significant environmental changes at the site were registered sedimentologically, geochemically, and palynologically by the episodic alternations between peat and inorganic sediments. Peat, dominated by *Rhizophora* and *Laguncularia* (white mangrove) pollen [32] indicates low water levels and a wetland environment, with salinity levels affected by the seepage of marine water through the barrier. On the other hand, the inorganic sediments, characterized by clay rich in Ti and the regional pollen signal, suggest higher backbarrier water levels and the transformation of the site from a wetland to an open-water lagoon, driven by an increase in precipitation and fluvial discharge to the basin. In core 5, seven such clay bands occurred during the past 4500 years, indicating episodes of wet climate and lagoon phases existing at approximately 4430–4270, 4080, 3950, 3680–3490, 3170–3080, 2990–2870, and 1680–0 years BP. The timing of these wet periods corresponds well with paleoclimatological evidence from the nearby coastal Laguna Tetitlan [34] and the Middle American Trench, located offshore of Oaxaca [35]. These abrupt sedimentary transitions are recorded for matching temporal intervals in cores 2, 3, 4, and 6. Distinct peat/clay transitions occurred at approximately 4500, 4300, and 1900 years BP in core 2, at 4600 years BP in core 3, and at 950 years BP (inferred) in core 6. It should be noted that not all six oscillations recorded in core 5 occur in all cores, and some undated, less distinct organic/inorganic transitions are present in other cores (e.g., the three LOI peaks in the lower half of core 6). The lithological differences among the six cores can be explained by the sites’ different sensitivities in recording water level changes, which are a function of water depth, sediment supply, sedimentation rate, and local habitat and vegetation (e.g., mangrove swamp versus mud flat), all of which vary spatially. Nevertheless, the onset of several of these clay layers, especially the ~4500 years BP event, indicating lagoon phases or wet episodes, are broadly synchronous among cores. It is also remarkable that the uppermost sediments in all six cores are characterized by lagoonal clay, which certainly represents the limnic deposits formed in the depositional environment of the modern shallow lagoon. Radiocarbon dates and inferred ages obtained from cores 2, 5, and 6 suggest that the modern lagoon phase has existed continuously for the last 1900 years.

While we had considered the possibility that the clay intervals in Laguna Mitla result from land movements attributable to either sudden subsidence or uplift from earthquakes or slow-slip events covering longer time periods, this notion has been rejected due to a lack of sedimentological evidence of sudden land movements (e.g., erosive contacts) in the cores. In addition, geochemical evidence of marine incursions is notably lacking; instead, the clay intervals are characterized by the enrichment of terrigenous chemical elements such as Ti. Along the coast of Guerrero, documented land movements from earthquakes range from ~7–15 cm [11] for coseismic uplift and 5–14 cm for slow slip events [36], insufficient to explain the formation of ~1 m deep lagoons, nor their relatively sudden transformation into mangrove-dominated swamps or mud flats.

### Sedimentary processes and provenances

**Fig 4:**
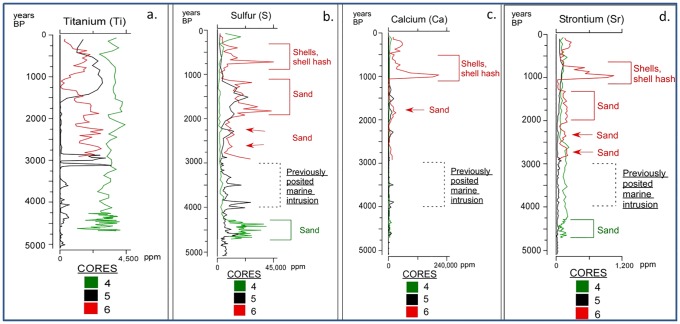
Elemental concentrations of sediment cores. Titanium, sulfur, calcium, and strontium concentrations vs age for seaward core 5 (black), and landward cores 4 (green) and 6 (red). Titanium concentrations (a.) are higher in core 4, nearest the input stream, than cores 5 and 6, farther east. Concentrations of sulfur (b.), calcium (c.), and strontium (d.) are generally higher in landward cores 4 and 6, than the seaward core 5. Spikes in marine elements are largely synonymous with shells (whole, hash) and/or sand, all largely absent in core 5 with the exception of basal sediments. A major marine event was posited from two landward sites, marked by a star and square in Fig 1 [12, 14]. A matching event is absent in the cores from this study, with no sand occurring at any site from 3000–4000 years BP.

The distributions of the elemental concentrations display distinct spatial patterns. The concentrations of Ti, which is commonly applied to identify terrestrial sediments [37] are many times higher in surface sediments collected from the western sites (1, 2, 3, and 4) than the eastern sites (5 and 6) (Fig 3). This suggests that slopewash, associated with large precipitation events and delivered by the ephemeral stream located in the northwest edge of the study site, is the primary source of this element. This spatial relationship remains constant throughout our record as Ti concentrations are higher at all times in core 4 than in the eastern cores 5 and 6 (Fig 4).

Due to their high concentrations in seawater and marine sediments, Sr, S, and Ca are commonly used marine indicators. In Laguna Mitla, however, surface sample concentrations of these elements are higher in the landward sites than the seaward sites (Fig 3), with the highest concentrations of all three elements occurring at site 6, ~ 1 km landward of the lagoon’s edge and adjacent to Isla Magueyes. This suggests that these elements do not originate from marine material delivered over the beach barrier, but rather the clastic and carbonate materials eroded from the relict beach ridge in the center of the lagoon. This is further supported by the low Sr, S, and Ca concentrations in the modern beach sand (Fig 3). Many event types can erode and redeposit these materials into the area of the landward transect, including earthquakes, tropical cyclone rain events, increased runoff during climatologically wet periods, and changes in land use.

This spatial pattern extends throughout the entirety of our record. Concentration maxima for S, Ca, and Sr occur in the landward cores 4 and 6, especially in intervals dominated by shells (whole, hash) and/or sand (Fig 4). The spikes in elemental concentrations in the uppermost sections of landward cores 2 and 4 (Fig 2) are not matched by corresponding spikes in seaward cores 3 and 5. This indicates that the material was not transported from a seaward location by marine incursions. Possibly these spikes result from the presence of gastropods, or ancient marine materials eroded from Isla Magueyes and other relict beach ridge remnants in or adjacent to the lagoon.

### Revisiting geological evidence attributed to marine incursion events

Large seawater intrusions can be expected to transport coastal sediments (e.g., sand) and marine organisms (shells, diatoms, foraminifera) inland. Marine deposition can vary from isolated overwash lobes to large sand sheets, often tens of centimeters thick [38, 39]. Tsunami waves are highly dynamic and can be extremely powerful, capable of transporting large objects landward [40], and depositing sand and other fine materials many kilometers inland [41]. Common characteristics of tsunami deposits include landward and seaward layering [42], landward fining [5], upward fining [43], rip-up clasts [7], and basal erosion [44].

The occurrence of a large marine incursion at ~3400 years BP for the area has been suggested [12–14], based on evidence found in core ACA 04–06 extracted from wetlands 5 km behind the coast in the northwestern part of the lagoon (red star, Fig 1), and core ACA 03–02 extracted landward of Isla El Conejo and 4 km from the coast (red square, Fig 1). Core ACA 04–06 [12], otherwise dominated by mud and silt, includes a ~60 cm unit of fine to coarse sand that contains rip-up clasts and high concentrations of such marine indicator elements as Na (sodium), Sr, and Ca, as well as a fining-upward stratigraphy. The authors suggest that this layer resulted from a marine incursion associated with an extremely rapid rise in relative sea level. Due to the layer’s sedimentary characteristics, thickness, and distance from the coast, a large tsunami was suggested as the probable delivery mechanism [12]. The downward movement of this event, unlike the uplift normally associated with historical earthquakes along the Guerrero coast, is posited to have been the result of a megathrust event that ruptured the “entire coupled plate interface” [13]. The ~6 m core ACA 03–02 [14] contains a 93 cm thick interval containing marine diatoms, sponge spicules, shells, and high concentrations of marine elements (Na, Sr, S), posited as resulting from marine intrusion with an extrapolated date of ~3500 years BP. This core does not include a sand unit corresponding to that found in ACA 04–06 [12]. The interval posited as resulting from marine intrusion also contains a peat layer [14].

It would be expected that such a marine incursion would have deposited a clear sedimentary signal at our sites, located behind the modern beach ridge plain and ~4 km closer to the coast than the ACA core locations. However, except for the basal sediments, sand layers are absent in cores 2, 3, and 5. Although distinct sand layers do occur in cores 4 and 6, they are much thinner (~1–30 cm) than the posited event deposits in core ACA 04–06, and they fail to exhibit common characteristics of tsunami deposition, including erosional contacts and deposition of marine shells. The elevated concentrations of S, Sr, and/or Ca in the surface samples, and downcore in cores 4 and 6 most likely represent material eroded from nearby Isla Magueyes. Although fine sand occurs from 64–53 cm in core 1, and clay/silt from ~84–79 cm in core 2 (decrease in LOI), core chronologies and stratigraphic correlations indicate that these layers were deposited much more recently than 3400 years BP, as evidenced by a sample dated to 1990±30 ^14^C years BP (1940 years BP) taken from 30 cm below the clastic layer in core 2. Furthermore, these sedimentological signatures in the westernmost cores 1 and 2 are not apparent in the eastward located cores 3, 4, 5, and 6, and may therefore suggest erosion and redeposition of remnant beach ridge materials mixed with sediments deposited from the slopes. Our results therefore do not support the identification of a significant marine incursion at 3400 years BP [12]; nor do our findings show definitive evidence of a large megathrust earthquake resulting in significant coseismic subsidence at this time [13, 14]. The lack of definitive stratigraphic evidence of tectonic movement, and the coherent, progressive ecological succession displayed in the multi-proxy record from Laguna Mitla [32], instead support the scenario of relative tectonic stability during the Late Holocene, previously implied by the presence of alluvial and deltaic plains observed by Ramírez-Herrera and Urrutia-Fucugauchi [45].

The presence of shells may explain the high levels of marine elements, particularly Ca, a major component of both terrestrial and marine shells, in core ACA 03–02, extracted landward of Isla El Conejo [14]. Either Isla Magueyes or Isla El Conejo, or other relict beach ridges further inland, are possible sources of the sediments and marine diatoms, which are capable of attaching to sand grains [46].

The low, episodic spikes in S, Sr, and Ca for the seaward cores 1, 3, and 5 in this study may possibly be explained by the episodic breaching of Laguna Mitla’s outlet channel following large precipitation events. Such breaching, a common occurrence for barred inner-shelf lagoons, has been documented from the nearby Laguna Nuxco [15], and can result in the intrusion of marine waters until the breach is closed. Minoura and Nakaya [47] documented saltwater intrusions which, while not severe enough to deposit sediments, were capable of leaving a geochemical signature in the sediments. Given the height, width, and stability of the beach ridge we believe that the most likely sedimentary signature of seismic activity over the last ~5000 years would be increased slopewash following the loosening of sediments associated with violent earth movement rather than deposition of thick sediment layers several kilometers inland.

## Conclusions

The main objective of this paper is to investigate the sedimentological processes and the provenances of bottom sediments deposited in Laguna Mitla, a barred inner shelf lagoon located on Mexico’s Pacific coast, in order to detect the sedimentary signal of tsunami-generated marine intrusions. Notably, concentrations of elements often associated with marine sources (Sr, S, Ca) are highest at landward sites 4 and 6, >2 km from the coast. We attribute the high concentrations of these elements to the erosion of clastic and carbonate materials from remnant beach ridges located in the lagoon’s interior. The concentration gradient of Ti is related to the proximity of the main feeder stream, located in the northwestern edge of the site.

Previous researchers have posited a sudden rise in relative sea level ~3400 years BP, possibly tsunami-generated, based on sedimentary evidence from two cores extracted ~4–5 km inland, each containing thick clastic layers/sections rich in marine elements, one of which included larger-grained material (sand) [12–14]. However, no corresponding sand layers occur in our three seaward cores, located closer to the ocean and directly behind the modern beach ridge plain. Any marine incursion process that deposited a thick layer of sand in the back side of the lagoon can be expected to have deposited an even thicker layer of sand at our core locations in front of that site. The landward cores extracted near the two relict beach ridges in the middle of the lagoon did possess thick sand layers with high concentrations of S, Sr, Ca, suggesting that this material was not introduced into the site by marine incursion, but by alternative means, such as erosion and reworking of sediments from islands El Conejo and Magueyes, possibly from heavy precipitation events or wave action.

Our analysis therefore does not support the interpretation of a tsunami or other significant marine incursions at this site ~3400 years ago. We furthermore suggest that the presence of sand layers with elevated concentrations of marine elements are not reliable indicators of extreme event for barred inner shelf lagoons along Mexico’s Pacific coast. This study highlights the complexity of properly interpreting sedimentary data in this tectonically active coastal region, further indicating the necessity of extracting multiple cores with a large spatial coverage to assess sediment provenance and depositional processes to properly determine tsunami risk and long-term tectonic histories. The lack of evidence of a severe marine intrusion event about 3400 years BP suggests that return periods for regional tsunamis may be lower than previously interpreted. This finding has significant implications for hazard risk assessment for Pacific coastal regions of Mexico. Additional regional sedimentary studies must be undertaken to properly assess the long-term risk. Ideally, these studies would successfully integrate sedimentary analyses over relatively large areas to more fully understand the relevant depositional mechanisms, as a means of definitively identifying tsunami deposits.
